# Can Tc 99m DTPA be Used in Adult Patients in Evaluation of Relative Renal Function Measurement as the Reference Tc 99m DMSA Method?

**DOI:** 10.4274/MIRT.20.03

**Published:** 2011-04-01

**Authors:** Hülya Yalçın, Aynur Özen, Emel Ceylan Günay, İnci Aliç Özaslan, Cahit Özer

**Affiliations:** 1 Mustafa Kemal University School of Medicine, Department of Nuclear Medicine, Hatay, Turkey; 2 Vakif Gureba Hospital, Department of Nuclear Medicine, İstanbul, Turkey; 3 Mersin University School of Medicine, Department of Nuclear Medicine, Mersin, Turkey; 4 Bagcilar Hospital, Department of Nuclear Medicine, İstanbul, Turkey; 5 Mustafa Kemal University School of Medicine Department of Family Medicine, Hatay, Turkey

**Keywords:** Relative renal function, Tc 99m DTPA, Tc 99m DMSA, adults

## Abstract

**Objective:** In the literature, there are many reports comparing relative renal function calculated with Tc 99m DTPA and Tc 99m DMSA in adults and children. However, there is no consensus about the results. As there is indeterminacy in the reliability of Tc 99m DTPA for the calculation of the relative renal functions, we retrospectively designed a study to compare the relative renal functions measured with Tc 99m DMSA and Tc 99m DTPA in adult patients with renal diseases

**Material and Methods:** We retrospectively analyzed the data of 144 patients who applied to Nuclear Medicine Department of three hospitals between 2009 and 2010 and who had both dynamic and static renal imaging. Renal dynamic scintigraphies were compared to the relative function measured using Tc 99m DMSA static scintigraphy. Comparison of relative renal function measurements using dynamic and static renal scintigraphies was performed using Pearson correlation test. The comparison results were expressed with Bland-Altman analysis.

**Results: ** The study was conducted with 144 patients and 288 kidneys. Fifty six of patients were male. Mean age was 39.9±15.2 years. Thirty four patients had hydronephrosis, 28 pyelonephritis, 53 renal calculi, 3 chronic renal failure, 2 acute renal failure, 1 benign renal neoplasia, 15 renal atrophy, 8 ureteropelvic junction stenosis. Relative renal function was calculated in Tc 99m DMSA and 99m Tc-DTPA studies. The mean relative renal functions measured with Tc 99m DTPA was 52.54±23.09% and 47.25±23.09, with Tc 99m DMSA 52.85±21.80% and 47.07±21.77% for right and left kidneys, respectively. In bivariate correlation analysis (Pearson) a significant positive correlation was found between the relative renal functions calculated with Tc 99m DTPA and Tc 99m DMSA (r =0.937, p< 0.001). In Bland-Altman plots, the mean difference between two methods was 0.3 and the correlation limits were between 16.2 to -15.5.

**Conclusion: ** As a result, we concluded that Tc 99m DTPA is also a good method for the relative renal function evaluation when compared to Tc 99m DMSA scan. Although Tc 99m DMSA is the most reliable method for the calculation of relative renal function, Tc 99m DTPA can be another choice for the calculation of relative renal function without a complementary DMSA scan particularly in patients who require renogram curve and GFR calculations.

**Conflict of interest:**None declared.

## INTRODUCTION

To measure the relative renal function, renal scintigraphy has been used for a long time. Different radiopharmaceuticals such as technetium-99m dimercaptosuccinic acid (Tc 99m DMSA), technetium- 99m diethylenetriamine pentaacetic acid (Tc 99m DTPA), technetium-99m mercaptoacetyltriglycine (Tc 99m MAG3), iodine 131 orthoiodohippurate (OIH) and more recently technetium-99m ethylenedicysteine (Tc 99m EC) were used (1). All of them can be used accurately to measure the relative renal function, although there are some differences among these radiopharmaceuticals (2) These differences are due to distinct biological properties of radiopharmaceuticals such as mechanisms of renal excretion, renal cell retention of radioactive material, level of plasma-protein bound and level of plasma clearance. However, Tc 99m DMSA as a static renal agent is considered the most reliable method to measure relative renal function (3,4,5) and the most appropriate tracer for renal cortical imaging (6). Tc 99m DMSA binding level to protein in mammals is 90%, this binding prevents significant glomerular filtration and Tc 99m DMSA primarily enters the kidney via peritubular extraction (7). It is primarily used in humans for cortical imaging and estimation of functional renal mass (8,9,10). Applications in humans include detection of pyelonephritis (11) and renal scars (12,13). Tc 99m DTPA is used for glomerular filtration rate (GFR) evaluation in mammals because no tubular secretion or reabsorption is observed but it is completely filtered by the glomerulus (8,10,14) In the literature, there are many reports comparing relative renal function calculated with Tc 99m DTPA and Tc 99m DMSA in adults and children. However, there is no consensus about the results and no study investigating the relative renal function calculation only in adults. In some papers, it is emphasized that relative renal function calculated with Tc 99m DTPA is as reliable as Tc 99m DMSA (15,16). On the other hand, in some of the studies, it is mentioned that Tc 99m DTPA is not as good as Tc 99m DMSA in relative renal function calculation (17,18) As there is indeterminacy in the reliability of Tc 99m DTPA for the calculation of relative renal function and lack of any study related to relative renal function calculation only in adults; we retrospectively designed a study to compare the relative renal functions measured with Tc 99m DMSA and Tc 99m DTPA in adult patients with renal diseases.

## MATERIALS AND METHODS

We retrospectively analyzed a total of 144 patients who applied to the Nuclear Medicine Department of three hospitals between 2009-2010 and who had both dynamic and static renal imaging. Renal dynamic scintigraphies performed with Tc 99m DTPA were compared to the relative function measured using Tc 99m DMSA static scintigraphy. There was at least 2 days between two studies and not more than a week. The data analyses were done in each hospital by a nuclear medicine physician.Tc 99m DTPA dynamic images were acquired with the patient in supine position and the detector of gamma camera placed at the posterior. The cameras [Symbia S (Siemens, Germany), E-cam (Siemens, USA), Brightview (Philips,USA)] were equipped with an all-purpose, low energy, parallel-hole collimator. All patients were injected with 259-370 MBq of Tc 99m DTPA, and dynamic images were recorded in a 128 x 128 matrix format every second for 1 minute and every 30 seconds for 20 minutes. Relative renal function was measured in a composite image (1 to 3 minutes after the injection). Renal and semilunar background regions of interest (ROIs) were drawn manually by a nuclear medicine physician. Tc 99m DMSA static images were also acquired with the patient in supine position. The camera was also equipped with an all-purpose, low energy, parallel-hole collimator. All patients were injected with 185 MBq of the radiopharmaceutical and static images were acquired in 256x256 matrix after 4 hours in the anterior, posterior, left and right posterior oblique, left and right lateral projections (250 kcounts/view or 5 minutes/view). Relative renal function was measured by drawing ROIs of each kidney in the anterior and posterior image with background correction made by drawing a perirenal background around each kidney by a nuclear medicine physician and relative renal function was calculated using the geometric mean method. Comparison of relative renal function measurement using Tc 99m DTPA dynamic renal scintigraphy and Tc 99m DMSA static scintigraphy was performed by Pearson correlation and Kruskal Wallis test. The comparison results were expressed with Bland-Altman analysis. The statistical analysis was carried out using the Statistical Package for the Social Sciences (SPSS) version 15 (SPSS, Chicago, IL). 

## RESULTS

**Figure 1:**
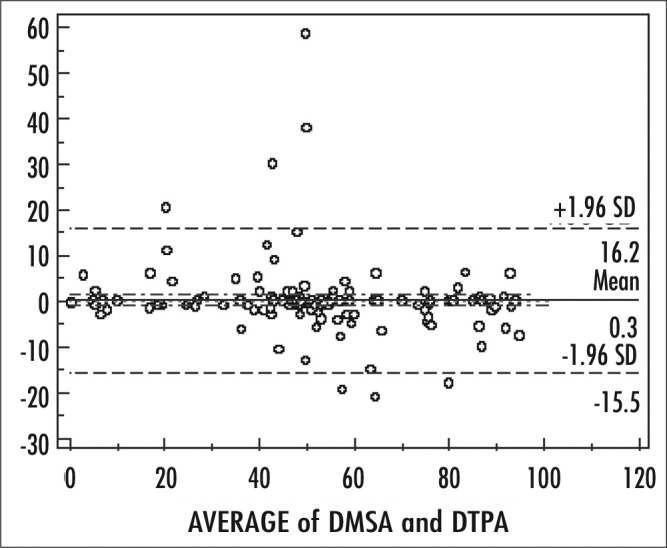
Bland-Altman analysis between Tc 99m DMSA and Tc 99m DTPA

The study was conducted with 144 patients from three hospitals retrospectively. The hospital charts of 144 patients were reviewed and the results of 288 kidneys were analyzed. Fifty six of patients were male and eighty eight female. The age range was between 18 and 81, mean age was 39.9±15.2 years. Thirty four patients had hydronephrosis, 28 pyelonephritis, 53 renal calculi, 3 chronic renal failure, 2 acute renal failure, 1 benign renal neoplasia, 15 renal atrophy, 8 ureteropelvic junction stenosis.Relative renal function measured with Tc 99m DMSA and 99m Tc-DTPA was calculated. The mean relative renal functions measured with Tc 99m DTPA was 52.54±23.09% and 47.25±23.09%, with Tc 99m DMSA 52.85±21.80% and 47.07±21.77% for right and left kidneys, respectively. In bivariate correlation analysis (Pearson) a significant positive correlation was found between the relative renal functions calculated with Tc 99m DTPA and Tc 99m DMSA (r=0.937, p<0.001). When the data were analyzed with Kruskal Wallis test according the patients diagnosis, we did not find differences between relative renal function calculated with both imaging methods (p=0.132 for right and p= 0.17 left kidney with Tc 99m DMSA, p= 0.212 for both kidneys with Tc 99m DTPA) (Table 1). In Bland-Altman plots, the mean difference between two methods was 0.3 and the correlation limits were between 16.2 to -15.5. Some values were out of the range; these were mostly related to kidneys with lower split renal function in Tc 99m DMSA (Figure 1).

## DISCUSSION

As we mentioned before, there are many reports comparing the relative renal function calculation results of Tc 99m DTPA and Tc 99m DMSA. However in our literature search we could not find any reports comparing the results of the relative renal function measured with Tc 99m DTPA and Tc 99m DMSA in a group of adult patients only with various renal diseases. The previous studies were performed in group of patients including adult and pediatric patients.

Taylor and his co-workers investigated the correlation between the relative renal uptake of Tc 99m DMSA and the relative glomerular filtration rate (GFR) in ten patients with serum creatinine ranging from 0.3 to 2.5 mg/dl. They used two methods to determine the renal uptake of Tc 99m DTPA. These methods showed excellent correlation with each other and with relative DMSA uptake (19).

In 2001, Sarı and Serdengeçti compared relative renal functions obtained with Tc 99m DTPA and Tc 99m DMSA in 42 patients. Their study group consisted mainly of children with a mean age of 10,5 years. They concluded that there were no statistical differences between relative renal function calculated with Tc 99m DTPA and Tc 99m DMSA. However, Tc 99m DMSA, as it is less expensive and easy to perform, could be an appropriate method to calculate relative renal function (15). 

On the other hand, in 2006, Domingues and his co-workers performed a study with 111 patients with age ranging between 0.17 to 79 years. Fifty five of patients had dynamic renal scintigraphy with Tc 99m DTPA and 56 with Tc 99m EC. All patients were imaged with Tc 99m DMSA. The relative renal functions calculated with Tc 99m DTPA and Tc 99m EC was compared with the results of Tc 99m DMSA. They concluded that relative renal function measured with Tc 99m EC was comparable to Tc 99m DMSA results, but the results of relative renal function measured with Tc 99m DTPA were statistically different (17).

In 2010, Lee and his colleagues calculated the relative renal function in 18 rabbits with unilateral ureteral obstruction. All the rabbits were imaged with Tc 99m DMSA or Tc 99m DTPA or Tc 99m MAG-3. The relative renal function was calculated and they found that although there were differences between left and right kidneys, no statistical differences were observed between groups. So, they concluded that dynamic renal imaging agents (Tc 99m DTPA and Tc 99m MAG-3) can be used to measure the relative renal function in place of the static image of Tc 99m DMSA (16).

In our study, we compared relative renal functions obtained with Tc 99m DTPA and Tc 99m DMSA in 144 adult patients (288 kidneys). The mean age was 39.9±15.2 years. We found a positive correlation between relative renal function calculated with Tc 99m DTPA and Tc 99m DMSA (r=0.937, p<0.001). Our study group consisted mostly of patients with diseases in which GRF and renogram curves are were as important as relative renal function. 

Even Tc 99m DMSA is an inexpensive and easy method20 used for cortical morphology and renal scar evaluation, patients who need the GFR and renogram curve results, the relative renal function calculated with Tc 99m DTPA may be used instead of static renal imaging with Tc 99m DMSA, since the comparison of the relative renal function results of Tc 99m DMSA and Tc 99m DTPA shows no statistical difference. There were some values out of range in Bland-Altman plots. These kidneys were mostly atrophic or hydronephrotic kidneys. In correlation to literature results, we thought that the difference between two methods was related to depth and location of the renal tissue (21,22).

**Study Limitations**

In multicenter studies, reproducibility analysis should be performed. In other words, the differences of relative renal function calculations with Tc 99m DMSA may have variability in different centers. However, in human studies this kind of analysis is practically difficult and ethically problematic. Because of the reasons mentioned above, we were not able to make a definition of reproducibility analysis in the current study.

As a result, we concluded that Tc 99m DTPA is also a good method for the relative renal function evaluation when compared to Tc 99m DMSA scan. Although Tc 99m DMSA is the most reliable method for the calculation of relative renal function, Tc 99m DTPA can be another choice for the calculation of relative renal function without a complementary DMSA scan particularly in patients who require renogram curve and GFR calculations.

## Figures and Tables

**Table 1:**
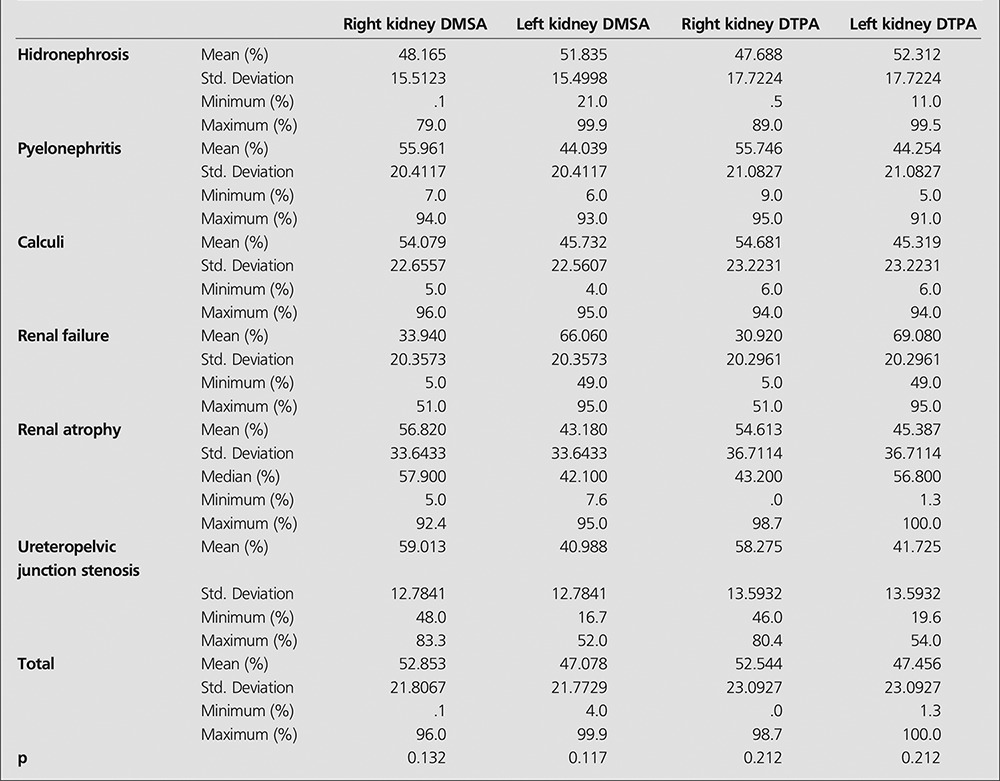
The mean, maximum and minimum (%) values of relative renal function calculated for each
